# Supplementary material for: Orthogonal cytokine engineering enables novel synthetic effector states escaping canonical exhaustion in tumor-rejecting CD8+ T cells
Source: Nat Immunol. 2023 Apr 20;24(5):869–83. doi: 10.1038/s41590-023-01477-2 (PMC10154250; doi:10.1038/s41590-023-01477-2)
Supplement: Supplementary file 1 — Reporting Summary [file 41590_2023_1477_MOESM1_ESM.pdf]

Corresponding author(s): George Coukos/Jesus Corria-Osorio

Last updated by author(s): Feb 23, 2023

Reporting Summary

Nature Portfolio wishes to improve the reproducibility of the work that we publish. This form provides structure for consistency and transparency in reporting. For further information on Nature Portfolio policies, see our Editorial Policies and the Editorial Policy Checklist.

Statistics

For all statistical analyses, confirm that the following items are present in the figure legend, table legend, main text, or Methods section.

n/a	Confirmed	
☐	☒	The exact sample size (n) for each experimental group/condition, given as a discrete number and unit of measurement
☐	☒	A statement on whether measurements were taken from distinct samples or whether the same sample was measured repeatedly
☐	☒	The statistical test(s) used AND whether they are one- or two-sided<br>*Only common tests should be described solely by name; describe more complex techniques in the Methods section.*
☒	☐	A description of all covariates tested
☐	☒	A description of any assumptions or corrections, such as tests of normality and adjustment for multiple comparisons
☐	☒	A full description of the statistical parameters including central tendency (e.g. means) or other basic estimates (e.g. regression coefficient) AND variation (e.g. standard deviation) or associated estimates of uncertainty (e.g. confidence intervals)
☐	☒	For null hypothesis testing, the test statistic (e.g. F, t, r) with confidence intervals, effect sizes, degrees of freedom and P value noted<br>*Give P values as exact values whenever suitable.*
☒	☐	For Bayesian analysis, information on the choice of priors and Markov chain Monte Carlo settings
☒	☐	For hierarchical and complex designs, identification of the appropriate level for tests and full reporting of outcomes
☐	☒	Estimates of effect sizes (e.g. Cohen's d, Pearson's r), indicating how they were calculated

Our web collection on statistics for biologists contains articles on many of the points above.

Software and code

Policy information about availability of computer code

Data collection	Flow cytometry data were collected on LSR II instruments with FACSDiva software v8.0.1 (BD)
Data analysis	Flow cytometry data was analyzed on FlowJo v10.7.1 (TreeStar) and statistical analysis performed on Prism v9.0 (GraphPad). scRNA-seq data was processed using the 10x Cell Ranger Pipeline. Genomic analyses were performed using R Studio v4.1 using the following packages : Seurat v4.1, scGate, ProjecTILs v2.0, clusterProfiler v3.12, scRepertoire , dynverse, GREIN(http://www.ilincs.org/apps/grein/?gse), GENAVI (https://junkdnalab.shinyapps.io/GENAVi/), SCENIC, ropls R package, AUCell R package, Webgestalt (http://www.webgestalt.org/) and pheatmap.<br><br>Incucyte images were quantified with the IncuCyte ZOOM integrated analysis software. The R script (FirstBatch_comb_TIL_B16_AllSamples.Rmd) to fully reproduce Fig. 2 is available here (https://github.com/carmonalab/GEEP_Jesus_Nov2021). The rest of our custom R scripts are available upon request.

For manuscripts utilizing custom algorithms or software that are central to the research but not yet described in published literature, software must be made available to editors and reviewers. We strongly encourage code deposition in a community repository (e.g. GitHub). See the Nature Portfolio guidelines for submitting code & software for further information.

Data

Policy information about availability of data

All manuscripts must include a data availability statement. This statement should provide the following information, where applicable:

- Accession codes, unique identifiers, or web links for publicly available datasets
- A description of any restrictions on data availability
- For clinical datasets or third party data, please ensure that the statement adheres to our policy

The scRNAseq data generated in this study are deposited in the National Center for Biotechnology Information Gene Expression Omnibus under accession number GSE200535. All other data are present in the article and supplementary files as well as the processed Seurat R object used for the analyses shown in Figs. 3-8 and the TIL_ACT reference map are available in the Figshare repository, as part of the Source data provided with this paper.

Publicly available files corresponding to the GEO accession codes: GSE126974, GSE123139, GSE99254, GSE125881 were obtained from the TISH repository(http://tisch.comp-genomics.org). The dataset EGAS00001004809 was obtained from EGA (European Genome-phenome Archive). Finally, the dataset E-MTAB-11773 was obtained from ArrayExpress and the dataset GSE206739 directly from the GEO repository .

Field-specific reporting

Please select the one below that is the best fit for your research. If you are not sure, read the appropriate sections before making your selection.

☒ Life sciences ☐ Behavioural & social sciences ☐ Ecological, evolutionary & environmental sciences

For a reference copy of the document with all sections, see nature.com/documents/nr-reporting-summary-flat.pdf

Life sciences study design

All studies must disclose on these points even when the disclosure is negative.

Sample size	No statistical methods were used to pre-determine sample size, but our sample sizes (4-10 mice/group) are similar to those reported in our previous publication (https://doi.org/10.1158/2159-8290.CD-21-0003).
Data exclusions	No data were excluded from analyses
Replication	All experiments presented in this study were performed using at least 2 biological or technical replicates. All presented results were confirmed in at least two independent experiments, unless otherwise noted. Specifically for scRNA-seq experiments were performed one time and combined 5 mice (biological replicates) per sample pool.
Randomization	On day 11 post tumor inoculation (average tumor volume 100-200 mm3) mice were randomly regrouped in order to have comparative average tumor volumes between experimental arms.
Blinding	Only for the anti tumor efficacy experiments mice were monitored three times/week by an independent investigator in a blinded manner

Reporting for specific materials, systems and methods

We require information from authors about some types of materials, experimental systems and methods used in many studies. Here, indicate whether each material, system or method listed is relevant to your study. If you are not sure if a list item applies to your research, read the appropriate section before selecting a response.

Materials & experimental systems

n/a	Involved in the study	
☐	☒	Antibodies
☐	☒	Eukaryotic cell lines
☒	☐	Palaeontology and archaeology
☐	☒	Animals and other organisms
☒	☐	Human research participants
☒	☐	Clinical data
☒	☐	Dual use research of concern

Methods

n/a	Involved in the study	
☒	☐	ChIP-seq
☐	☒	Flow cytometry
☒	☐	MRI-based neuroimaging

Antibodies

Antibodies used	FCR blocking reagent (clone 2.4G2) (1:50) BD Biosciences Cat#553141<br>PE/Cy5.5 anti-mouse CD3e (clone 2. 145-2C11) (1:50) Invitrogen Cat# 14-0031-82<br>Brilliant Violet 711 anti-mouse CD8a (clone 2. 53-6.7) (1:50) Biolegend Cat# 100748

PE/Dazlee 594 anti-mouse CD45.1 (clone A20) (1:100) Biolegend Cat# 110748
PE-mouse anti-human IgG4 (clone HP6025) (1:100) abcam Cat#99825
PE-anti-mouse IL-33 (clone 396118) (1:50) Invitrogen Cat#MA5-23640
Purified polyclonal anti-mouse PD-1 (final conc. 2ug/mL) R&D Cat#AF1021
HRP anti-human IgG4 pFc’ (clone: HP6023) (1:2000) abcam Cat#ab99817
Purified polyclonal anti-human IL-2 Antibody (final concentration 3 ug/mL) R&D Cat#AF-202-SP
Biotin Polyclonal anti-human IL-2 Antibody (1:500) Invitrogen Cat# 13-7028-81
PE/Dazlee 594 anti-mouse CD4 (clone GK1.5) (1:100) Biolegend Cat# 100456
PE- anti-mouseFOXP3 (clone FJK-16s) (1:50) Invitrogen Cat# 12-5773-82
Brilliant Violet421 - anti-mouse/human CD44 (clone IM7) (1:100) Biolegend Cat# 103040
APC/Cy7-anti-mouse PD-1 (clone 29F.1A12) (1:100) Biolegend Cat# 135224
BV605- anti-mouse Ly-6C (clone HK1.4) (1:800) Biolegend Cat# 128036
APC- anti-mouse Granzyme C (clone SFC1D8) (1:100) Biolegend Cat# 150812
PE/Cy7- anti-mouse Granzyme C (clone SFC1D8) (1:100) Biolegend Cat# 150804
Rabbit anti TCF1 (TCF7) antibody (clone C63D9) (1:200) Cell Signaling Technology Cat# 2203S
PE-anti-rabbit IgG (H+L), F(ab')2 Fragment (1:250) Cell Signaling Technology Cat# 8885S
Alexa Fluor 488-anti-rabbit IgG (H+L), F(ab')2 Fragment (1:250) Cell Signaling Technology Cat# 4408S
PE/Cy7- anti-mouse/human Granzyme B (clone CLB-GB11) (1:50) Novus Biological Cat# NBP1-50071PECY7
AF647- anti-mouse/human Granzyme B (GB11) (1:50) Biolegend Cat# 515406
PE/Cy7- anti-mouse 4-1BB (clone 17B5) (1:50) Invitrogen Cat# 25-1371-82
Brilliant Violet 605 anti-mouse CD25 (clone PC61) (1:200) Biolegend Cat# 104530
Brilliant Violet 605 anti-mouse CD69 (clone H1.2F3) (1:100) Biolegend Cat# 104530
Brilliant Violet 421 anti-mouse CD69 (clone H1.2F3) (1:100) Biolegend Cat# 104528
Brilliant Violet 605 anti-mouse/human CD44 (clone IM7) (1:100) Biolegend Cat# 103047
PecCP-Cy5.5 anti-mouse/human CD44 (clone IM7) (1:100) Biolegend Cat# 103032
Brilliant Violet 421 anti-mouse TIM-3(clone RMT3-23) (1:50) Biolegend Cat# 119723
PE/Cy7 anti-mouse/human KLRG1(clone 2F1/KLRG1) (1:100) Biolegend Cat# 138416
Brilliant Violet 650 anti-mouse CX3CR1 (clone S011F11) (1:300) Biolegend Cat# 149033
Brilliant Violet 605 anti-mouse Ki-67(clone 16A8) (1:100) Biolegend Cat# 652413
PerCP-Cyanine5.5- anti-mouse IFNg (clone XMG1.2) (1:50) Invitrogen Cat# 45-7311-82
PE/Cy7- anti-mouse TNFa (clone MP6-XT22) 1:100 Biolegend Cat# 506324
BV650- anti-mouse TNFa (clone MP6-XT22) 1:100 Bdbioscience Cat# 563943
PE/Cy7- anti-mouse CD62L (clone MEL-14) (1:100)Biolegend Cat# 104418
PE- anti-TOX antibodies human and mouse (clone REA473) (1:50) Miltenyi Cat# 130-107-785
APC- anti-TOX antibodies human and mouse (clone REA473) (1:50) Miltenyi Cat# 130-107-784
APC- Armenian Hamster IgG Isotype Ctrl Antibody (clone HTK888) (1:100) Biolegend Cat# 400912
PE/Cy7- Armenian Hamster IgG Isotype Ctrl Antibody (clone HTK888) (1:100) Biolegend Cat# 400922
PE- GzmA (1:100) Biolegend Cat# 149704
APC-Prf1 (1:100) Biolegend Cat# 154304
Purified anti-mouse CD8a Antibody Biolegend Cat#100702
Purified anti-mouse CD105 Antibody Biolegend Cat#120402
Purified anti-mouse CD45.1 Biotin (clone A20.1) In house -
Cy™3 AffiniPure Donkey Anti-Rabbit IgG (H+L) Jackson ImmunoResearch Cat#711-165-152
Alexa Fluor 488 Donkey anti-Rat IgG (H+L) Highly Cross-Adsorbed Secondary Antibody Invitrogen Cat#A21-208
InVivoMAb anti-mouse CD4 (Clone: GK1.5) BioXcell Cat#BE0003-1
InVivoMAb anti-mouse PD-L1 (Clone: 10F.9G2) BioXcell Cat#BE0101
InVivoMAb anti-mouse TIM-3 (Clone: RMT3-23) BioXcell Cat#BE0115
Rat anti mouse anti-ST2 antibody was used (Clone : DJ8mdbioproducts CatNumber: 101001PE, dilution 1:50)
PE Rat IgG1, λ Isotype Ctrl Antibody Biolegend CAT# 401906
anti mouse CD3 Monoclonal Antibody (17A2), (final conc. 1 ug/mL) eBioscience™ CAT# 14-0032-82

Validation

Antibodies were validated by the manufacturer.

Eukaryotic cell lines

Policy information about cell lines

Cell line source(s)	The MC38-OVA tumor cell line was obtained from Pedro Romero's Lab (UNIL). B16-F10 (CRL-6475) tumor cell lines was purchased from ATCC and retroviral transduced to express ovalbumin (B16-OVA). Both were grown as a monolayer in DMEM supplemented with 10% fetal calf serum (FCS), 100 U/mL of penicillin, and 100 ug/mL streptomycin sulphate. The Phoenix Eco retroviral ecotropic packaging cell line, derived from immortalized normal human embryonic kidney (HEK) cells was obtained from ATCC (CRL-3214) and maintained in RPMI 1640-Glutamax media supplemented with 10% heat-inactivated FBS, 100 U/ml penicillin and 100 g/mL streptomycin sulfate.
Authentication	Cell lines were not authenticated after purchase from ATCC
Mycoplasma contamination	All cell lines were tested for mycoplasma contamination prior to use in experiments
Commonly misidentified lines (See ICLAC register)	NA

Animals and other organisms

Policy information about studies involving animals; ARRIVE guidelines recommended for reporting animal research

Laboratory animals	6 weeks-old female C57BL/6 mice aged were purchased from Harlan (Harlan, Netherlands) and housed at the animal facility at the University of Lausanne in compliance with guidelines. C57BL/6 OT-1 CD45.1+ and C57BL/6 Pmel were obtained from Pedro Romero's Lab (UNIL). All in vivo experiments were conducted in accordance and with approval from the Service of Consumer and Veterinary Affairs (SCAV) of the Canton of Vaud, Switzerland under the license VD3526.
Wild animals	Study did not involve wild animals
Field-collected samples	Study did not involve samples collected in the field
Ethics oversight	Service de la consommation et des affaires vétérinaires (SCAV) https://www.ge.ch/organisation/service-consommation-affaires-veterinaires

Note that full information on the approval of the study protocol must also be provided in the manuscript.

Flow Cytometry

Plots

Confirm that:

☒ The axis labels state the marker and fluorochrome used (e.g. CD4-FITC).

☒ The axis scales are clearly visible. Include numbers along axes only for bottom left plot of group (a 'group' is an analysis of identical markers).

☒ All plots are contour plots with outliers or pseudocolor plots.

☒ A numerical value for number of cells or percentage (with statistics) is provided.

Methodology

Sample preparation	Tumors were excised and dissociated into single-cell suspension by combining mechanical dissociation with enzymatic degradation of the extracellular matrix using the commercial Tumor Dissociation kit for mouse (Miltenyi, Cat# 130-096-730). Following single cell suspension, 2.5x106 live cells were seeded in 96-well plates and incubated with 50 ul of Live/Dead Fixable aqua dead for 30' in PBS at 25°C, then Fc receptors were blocked by incubation for 30 min. at 4°C with 50 ul of purified anti-CD16/CD32 mAb. Cells were then stained for 30 min at 4°C with the fluorochrome-conjugated mAbs of interest (see above) in 50 ul of FACS Buffer. Subsequently, the cells were washed twice, and fixed/permeabilized using the FoxP3 transcription factor staining buffer set for intracellular staining. Fluorescence minus one (FMO) controls were stained in parallel using the panel of antibodies with sequential omission of one antibody. Precision Count Beads™ (Biolegend, Cat# 424902) were used to obtain absolute counts of cells during acquisition on the flow cytometer.
Instrument	A BD FACSAriaIII instrument or a A BD FACSAriaII instrument were used for cell sorting and a BD LSR II instrument with FACSDiva software v8.0.1 to collect data for analysis.
Software	FACSDiva software v8.0.1(BD) was used on LSR II instrument for data collection; data was analyzed on FlowJo v10.7.1 (TreeStar) and statistical analysis performed on Prism v9.0 (GraphPad)
Cell population abundance	Sorted samples had purity > 95% as confirmed by re-sampling after sorting.

Gating strategy

FSC-A/SSC-A was used to gate on cells. Doublets were excluded through SSC-H/SSC-W and FSC-H/FSC-W. Dead cells were excluded with Live/Dead Fixable aqua. CD8 T cells were gated as CD8 positive. OT1 transferred cells were identified using the congenic marker CD45.1. A representative general gating strategy is depicted in Extended Data Figure 3a.

☒ Tick this box to confirm that a figure exemplifying the gating strategy is provided in the Supplementary Information.
